# Supplementary material for: Estimating the burden of foodborne gastroenteritis due to nontyphoidal Salmonella enterica, Shigella and Vibrio parahaemolyticus in China
Source: PLoS One. 2022 Nov 7;17(11):e0277203. doi: 10.1371/journal.pone.0277203 (PMC9639838; doi:10.1371/journal.pone.0277203)
Supplement: S1 Table — (DOCX) [file pone.0277203.s001.docx]

**S1 Table. The values behind the minimum, most likely and maximum values reported.**

| **Province** | **AGI**^a^ **incidence per person-year** | **Proportion of cases seeking medical care (%)** | **Proportion of cases submitting a stool sample for testing among those seeking medical care (%)** |
| --- | --- | --- | --- |
| Shanghai | 0.16 | 36.9 | 34.4 |
| Jiangsu | 0.63 | 45.0 | 38.7 |
| Zhejiang | 0.43 | 58.8 | 39.2 |
| Jiangxi | 0.77 | 76.8 | 49.9 |
| Guangxi | 0.48 | 63.4 | 15.6 |
| Sichuan | 0.73 | 57.8 | 27.2 |
| Total | 0.56 | 56.1 | 32.7 |

^c^AGI: Acute gastrointestinal illness.
