# Supplementary material for: Estimating the burden of foodborne gastroenteritis due to nontyphoidal Salmonella enterica, Shigella and Vibrio parahaemolyticus in China
Source: PLoS One. 2022 Nov 7;17(11):e0277203. doi: 10.1371/journal.pone.0277203 (PMC9639838; doi:10.1371/journal.pone.0277203)
Supplement: Attachment — Submitted filename: Response to Reviewers.docx [file pone.0277203.s003.docx]

PONE-D-21-34340

Estimating the burden of foodborne gastroenteritis due to nontyphoidal Salmonella enterica, Shigella and Vibrio parahaemolyticus in China

PLOS ONE

Journal Requirements:

Please remove any funding-related text from the manuscript and let us know how you would like to update your Funding Statement.

**Response: Accepted and removed funding-related text from the manuscript.**

**Funding Statement is as following:**

**This study was financially supported by the National Natural Science Foundation of China (No. 81673175), CAMS Innovation Fund for Medical Science (CIFMS 2019-I2M-5-024) and the Jiangsu Provincial Preventive Medicine Association (No. Y2018084).**

4. In your Data Availability statement, you have not specified where the minimal data set underlying the results described in your manuscript can be found. PLOS defines a study's minimal data set as the underlying data used to reach the conclusions drawn in the manuscript and any additional data required to replicate the reported study findings in their entirety. All PLOS journals require that the minimal data set be made fully available.

**Response: Accepted and revised the data availability as following:**

**Yes- all data are fully available without restriction.**

5. We note that Figure 1 in your submission contain [map/satellite] images which may be copyrighted.

We require you to either (1) present written permission from the copyright holder to publish these figures specifically under the CC BY 4.0 license, or (2) remove the figures from your submission:

**Response: Accepted and removed Figure 1.**

Reviewers' comments:

1. Page 5, Line 85

It would be better if you could be more specific in how you chose the population survey sentinel sites, especially what you mean by "appropriateness".

**Response: Accepted and revised as following:**

**The sentinel sites were chosen on the basis of their appropriateness (geographically representative of China, e.g., urban/rural, southern/northern) (L87)**

2. Page 8, Line 150

It might be better to explain the reason you used PERT distribution in your model. It would probably be helpful for the readers who are not so familiar with probability distribution.

**Response: Accepted and revised as following:**

**Considering the characteristics of the data, a PERT distribution with specified minimum, most likely and maximum values was used to model uncertainty for each proportion. (L151-152)**

3. Page 18, Line260-266

Authors have discussed that storage of seafood at room temperature in Chinese market might be the important factor to high Vibrio parahaemolyticus infection estimated and reported in China. It might be more intelligible to add "establishment of cold-chain" in the "effective measures by the government" discussion as one example if it is appropriate.

**Response: Accepted and added a sentence as following:**

**including the establishment of cold-chain (L269)**

4. General comment.

Same with other disease surveillance, use of burden of foodborne disease estimate results presented in this paper is most effective when compared with past estimates to see the changes through time, under same or similar data collection and calculation format. I wish the authors to continue their research.

**Response: Accepted and added a paragraph as following:**

**As with other disease surveillance, use of burden of foodborne disease estimate results presented in this paper is most effective when compared with past estimates to see the changes through time, under same or similar data collection and calculation format. (L305-307)**
