# Supplementary material for: Estimating the burden of foodborne gastroenteritis due to nontyphoidal Salmonella enterica, Shigella and Vibrio parahaemolyticus in China
Source: PLoS One. 2022 Nov 7;17(11):e0277203. doi: 10.1371/journal.pone.0277203 (PMC9639838; doi:10.1371/journal.pone.0277203)
Supplement: Attachment — Submitted filename: CBOD_Response to Reviewers_2.docx [file pone.0277203.s004.docx]

October 18, 2022

Dear Dr. James Mockridge,

We are very grateful for your evaluation of our paper entitled “Estimating the burden of foodborne gastroenteritis due to nontyphoidal *Salmonella enterica*, *Shigella* and *Vibrio parahaemolyticus* in China” (PONE-D-21-34340R2). The comments were exceptionally helpful. Below, we describe how we have addressed each of them in the revision. The comments are in italics, and our responses are in regular type.

**Reviewer #3’s Comments:**

*1-The response to one reviewer wanting a more detailed description of the PERT model included the comment by the investigators that, considering the characteristics of the data, a PERT distribution with specified minimum, most likely and maximum values was used to model uncertainty for each proportion. (L151-152). This was a poorly presented partial response to the larger concern about the general statistical presentation.*

**RESPONSE**. Thanks for raising this important issue. Your suggestions have greatly improved the paper. In the revised manuscript, we have clarified the PERT distribution as following.

PERT distributions with specified minimum, most likely and maximum values were used to model uncertainty for variables I_AGI_, P_1_, P_2_, and P_4_. (L149-151)

*2-Table 1 gives a good descriptive summary of some of the results including the PERT model. However, the detail of the third column (Data, formula, distribution) could be enhanced. Presumably, the PERT (A,B,C) inclusions assume that the A, B and C are these min, most likely, and max values mentioned above. Please correct this reviewer if I am in error. Otherwise, the last column appears to summarize the results being sought. The investigators have to pay attention to communicating these results in a way that is interpretable by a non statistical reader. The entire manuscript should be reviewed and edited to assure clarity of the statistical presentation.*

**RESPONSE**. We are grateful for these insightful questions. In the revised manuscript, we have added the following foodnote to Table 1.

^d^ PERT (*a*, *b*, *c*): PERT distribution with minimum value *a*, most likely value *b*, and maximum value *c*. (L166)

We would like to thank again the editors and reviewers for all the time and effort they put into evaluating and improving this manuscript. We are extremely grateful for this.
